# Molecular modeling analyses of functionalized cellulose

**DOI:** 10.1038/s41598-024-77629-7

**Published:** 2024-11-12

**Authors:** Hend A. Ezzat, Nayera M. El‑Sayed, Dina Shehata, Hanan Elhaes, Asmaa Ibrahim, Haitham Kalil, Medhat A. Ibrahim, Moataz M. Yousef, Ibrahim S. Yahia, Heba Y. Zahran, Islam Gomaa

**Affiliations:** 1 Nano Unite, Space Lab, Solar and Space Research Department, National Research Institute of Astronomy and Geophysics (NRIAG), Helwan, Cairo, 11421 Egypt; 2 Physics Department, Faculty of Science, Mansoura University, Mansoura, 35516 Egypt; 3 Physics Department, Faculty of Women for Arts, Science and Education, Ain Shams University, Cairo, 11757 Egypt; 4 Chemistry Department, Faculty of Science, Suez Canal University, Ismailia, 41522 Egypt; 5 Spectroscopy Department, National Research Centre, 33 El-Bohouth St., Dokki, Giza, 12622 Egypt; 6 Molecular Modeling and Spectroscopy Laboratory, Centre of Excellence for Advanced Science, National Research Centre, 33 El-Bohouth St., Dokki, Giza, 12622 Egypt; 7 Nanotechnology Research Centre (NTRC), The British University in Egypt (BUE), Suez Desert Road, El-Sherouk City, Cairo, 11837 Egypt; 8 Nanoscience Laboratory for Environmental and Bio-Medical Applications (NLEBA), Semiconductor Lab., Metallurgical Lab.1., Physics Department, Faculty of Education, Ain Shams University, Roxy, Cairo, 11757 Egypt

**Keywords:** Cellulose, Nanocomposite, GO, DFT: B3LYP/3-21 g**, ATR-FTIR, Environmental sciences, Physics

## Abstract

Functionalization of cellulose with nanomaterials and functional groups is essential for enhancing its properties for specific applications, such as flexible sensors and printed electronics. This study employs Hartree Fock (HF) and Density Functional Theory (DFT) calculations to investigate the vibrational spectra of cellulose, identifying DFT: B3LYP/3–21 g** as the optimal model aligning with experimental spectra. Using this model, we examined the impact of functionalizing cellulose with various groups (OH, NH_2_, COOH, CH_3_, CHO, CN, SH) and graphene oxide (GO) on its electronic properties. The results indicate that cellulose functionalized with GO (Cellulose-GO) has the lowest bandgap energy (0.1687 eV), and improvements in reactivity, stability, and electronic properties were confirmed through Molecular Electrostatic Potential (MESP) and Total Dipole Moment (TDM) analyses. The spectrum of Density of States (DOS) for the cellulose functionalized with different groups shows several peaks, indicating various energy levels where electronic states are concentrated. The Projected Density of States (PDOS) analysis reveals how different functional groups affect the electronic structure of cellulose. Moreover, the (Cellulose-GO) composite was characterized using an Attenuated Total Reflection Fourier Transform Infrared (ATR-FTIR) spectrometer, revealing interaction through the OH group of CH_2_OH, as indicated by a new band at 1710 cm^−1^, consistent with theoretical predictions. Overall, this study demonstrates that functionalization with GO enhances cellulose’s responsiveness, degradation, and electrical properties, making it suitable for applications in flexible electronic devices and protective barriers against corrosion.

## Introduction

Surface functionalization is an effective way to alter the properties of a material’s surface^1^. Recently, polymer surface functionalization has gained importance for managing the electronic characteristics and activity of polymer surfaces^2^. This is achieved by modifying the polymer with active functional groups and/or nanomaterials^3,4^. Advancing natural polymers through functionalization represents an important step in green technology^5^. Among natural polymers, conjugated polymers that can be processed with the appropriate solution are always among the sought-after candidates for inexpensive electronics and optoelectronics technology^6^. Additionally, organic molecule-based materials have become highly significant in materials science, particularly for applications in optoelectronic and mechanochromic luminescent devices^7^. These materials, with their conjugated electron systems, offer enhanced efficiency, stability, and flexibility, making them promising candidates for advanced research. In this context, functionalizing polysaccharides with various organic groups presents an opportunity to leverage these properties.

Polysaccharides, as the most important type of natural polymer, have efficient and active functional groups that perform a wide range of crucial functions^8^. Cellulose, the most well-known polysaccharide, is composed of D-anhydro glucopyranose units linked by -(1,4)-glycosidic bonds^9^. It is a widely available renewable resource, sourced from plants such as trees, cotton, and hemp, making it inexpensive to produce due to established and cost-effective extraction and processing methods^10^. Cellulose’s natural porosity makes it ideal for filtration and absorbent products like air filters, as well as for certain battery components^11^. Its ability to form thin films and sheets is essential for industrial uses, such as packaging materials, biodegradable plastics, photographic film bases, coatings, and membranes^12^. With high permittivity, cellulose can store and release electrical energy, making it suitable for electronic applications like capacitors^13^. Additionally, its chemical stability ensures durability and performance in diverse environments^14^. Thus, cellulose is utilized in a wide range of applications^15–18^ .

Natural fibrous materials have emerged as a practical alternative to synthetic fibers^19^. However, regenerated cellulose’s weak mechanical properties limit its use as a membrane material. To enhance its properties, other materials are often added to cellulose, resulting in composites with improved chemical and physical capabilities^20,21^. Functionalizing cellulose and its derivatives with graphene oxide (GO) enhances their thermal stability and ion transport^22,23^, making them suitable for applications like UV shielding and water purification^24,25^. Functional groups containing oxygen atoms can bind metal ions or polymers like cellulose to form metal complexes. Dispersion of nanoparticles in a polymer matrix improves mechanical, thermal, and electrochemical properties^26^. Each nanoparticle, such as GO, enhances different aspects of the polymer. Graphene, a two-dimensional sheet of sp2 bonded carbon, has unique visual, electrochemical, mechanical, and thermal properties^27^. Graphene oxide (GO), a chemically synthesized precursor of graphene, contains various oxygenated functional groups. These groups disrupt graphene’s conjugated structure, making GO an insulating material, but also improve interfacial interaction with substrates, significantly enhancing the physical properties of composite materials^28^.

Molecular modeling is a computational technique that simulates chemical structures and reactions numerically. Its primary role is to provide insights into molecules and reactions that are difficult to observe directly, such as transition states and unstable intermediates^29^. This method assesses the energy of molecular structures, optimizes their geometry, and calculates their vibrational frequencies^30^. It is widely used to generate reliable spectroscopic and structural data across various fields^31^. Semi-empirical methods, which use parameters from experimental results, simplify computations, offering cost-effective and reasonably accurate predictions of energy and structures when good parameters are available^32^. These methods are largely based on quantum mechanics and produce high-quality quantitative estimates for many systems^33^. Density Functional Theory (DFT) is similar to ab initio methods, requiring comparable computational effort as Hartree-Fock theory, the least expensive ab initio method. DFT calculations have been extensively used to investigate the electronic and photovoltaic properties of materials^34^. It is preferred for its inclusion of electron correlation effects, providing more accurate results than some ab initio methods^35^. Although DFT has limitations, such as issues with exchange interaction treatment and long-range noncovalent interactions, it is highly effective for small molecular systems^36^.

The primary goal of this research is to conduct vibrational computations for cellulose to identify the optimal basis set that aligns with experimental results. Theoretical calculations were performed using HF and DFT: B3LYP with various basis sets, including 3–21 g, 6–31 g, 6–311 g, LANL2DZ, and LANL2MB. The best basis set will then be used to determine the most suitable site for cellulose chain functionalization (center or terminal). Additionally, to study the impact of functionalization on cellulose’s electrical properties and bandgap variation, key properties such as total dipole moment, HOMO-LUMO energy gap, molecular electrostatic potential, and density of states (DOS) were calculated for these model molecules. Several functional groups, including (OH, NH_2_, COOH, CH_3_, CHO, CN, SH) and GO, were proposed for cellulose functionalization at the ideal interaction site.

## Materials and methods

### Materials

Pure microcrystalline cellulose powder (20 μm) was sourced from Sigma-Aldrich Company, Inc., USA, and dimethyl sulfoxide (DMSO) was obtained from Labscan Ltd., for film preparation. Graphene oxide (GO) was synthesized using graphite powder from Fluke, Germany, and solvents including H_2_SO_4_ (98%), H_2_O_2_ (30%), and HCl (33%), all purchased from El-Nasr Pharmaceutical Company in Egypt. Additionally, KMnO_4_ was acquired from Alfa Aesar (98%, Germany).

### Synthesis of GO

The Hummers method was used to synthesize GO^37^. In this process, 1 g of graphite was stirred with 35 ml of H_2_SO_4_ and 3 g of KMnO_4_ for about an hour in an ice-water bath, keeping the temperature below 20 °C. After an hour, approximately 105 ml of H_2_O_2_ was gradually added to the solution and heated to around 100 °C. The mixture was then diluted with 280 ml of distilled water. The resulting GO precipitate was washed with 2 M HCl, followed by water, and then dried.

### Preparation of cellulose and cellulose-GO

The casting method was employed to prepare cellulose films and cellulose films containing a significant amount of GO. For the cellulose film, 0.25 g of cellulose was dissolved in 100 mL of DMSO using a magnetic stirrer at 800 rpm for 2 h at 70 °C until fully dissolved, and then cast into a glass petri dish. For the Cellulose-GO film, 0.25 g of cellulose was dissolved in 100 mL of DMSO as before, and then 0.025 g (10% wt.) of GO was added. The mixture was stirred at the same temperature for 2 h or until the solution became homogeneous, and then dried in glass petri dishes.

### Fourier transform infrared spectroscopy

The Fourier Transform Infrared (FTIR) spectra were obtained using a Vertex 70 FTIR spectrometer manufactured by Bruker Optik GmbH, Germany. The instrument was equipped with a diamond ATR crystal system and operated in the spectral range of 4000 –400 cm^[-1^with a spectral resolution of 4 cm^[-1^.

### Calculation details

Optimization and vibrational calculations were conducted for Cellulose using HF^38^and DFT: B3LYP methods^39–41^, employing various basis sets such as 3–21 g, 6–31 g, 6–311 g, LANL2DZ, and LANL2MB. The DFT/B3LYP functional with various basis sets was chosen based on its established reliability and accuracy for organic compounds. This level of theory has been widely accepted in the literature for studying the electronic properties of polymers and functionalized materials. The combination of DFT and B3LYP has been shown to yield satisfactory results for predicting binding energies, electronic structures, and other critical properties relevant to our study. The Gaussian 09 software package from Gaussian, Inc., Wallingford, CT, USA, was utilized for structure optimization and molecular characteristic calculations at the Molecular Spectroscopy and Modeling Unit, National Research Centre, Cairo, Egypt^42^. Total Dipole Moment (TDM), bandgap energy, and Molecular Electrostatic Potential (MESP) were also determined for functionalized cellulose with different groups (OH, NH2, COOH, CH_3_, CHO, CN, SH) and graphene oxide (GO) using the DFT: B3LYP/3–21 g** model^43,44^. Moreover, various parameters indicative of inhibition efficiency was determined through DFT calculations to evaluate the inhibition enhancement resulting from functionalization with GO. The quantum parameters included electron affinity (EA = − ELUMO), ionization potential (IP = − EHOMO), global hardness (η = (IP − EA)/2), chemical softness (σ = 1/η), electronegativity (χ = (IP + EA)/2), fraction of transferred electrons (∆N = (χFe − χinh)/2(ηFe − ηinh), where χFe represents the electronegativity of iron (which is zero) and ηFe (which is 7 eV)), and electrophilicity index (ω)^45,46^. Chemical hardness and softness were also calculated. These parameters simulate the chemical reactivity and stability of structures.

## Results and discussion

### Molecular modeling study

**Figure 1:**
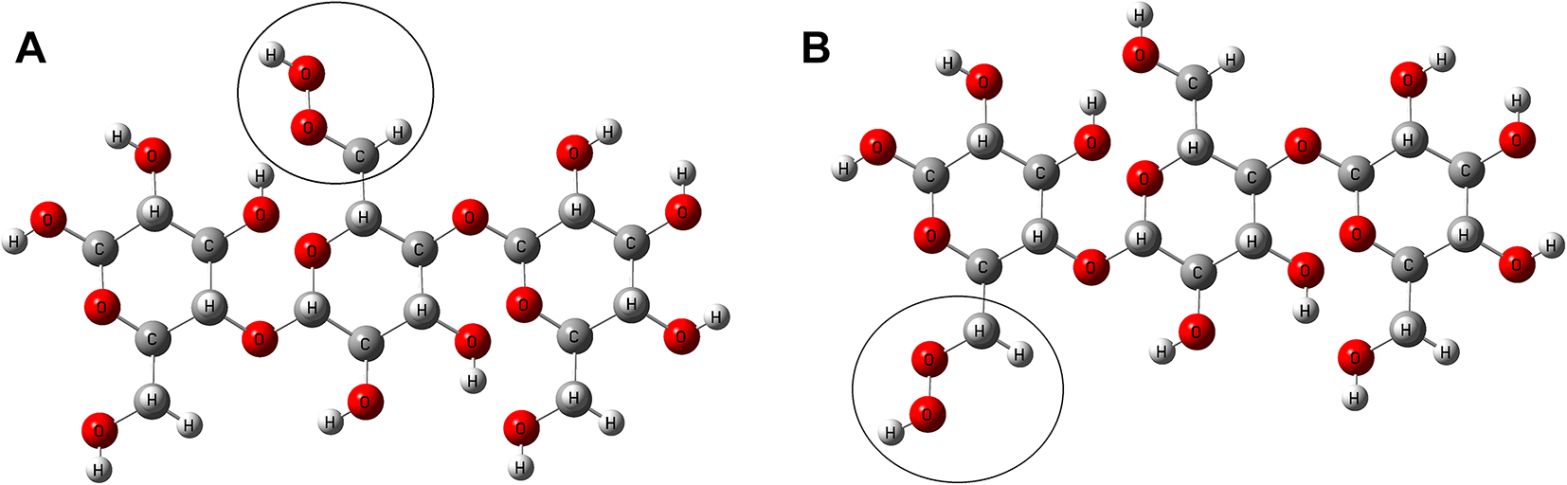
Optimized structure of a cellulose model consisting of three units, functionalized with an OH group at the active site of the CH 2 OH group in two different positions: (**a**) Center, and (**b**) Terminal.

**Figure 2:**
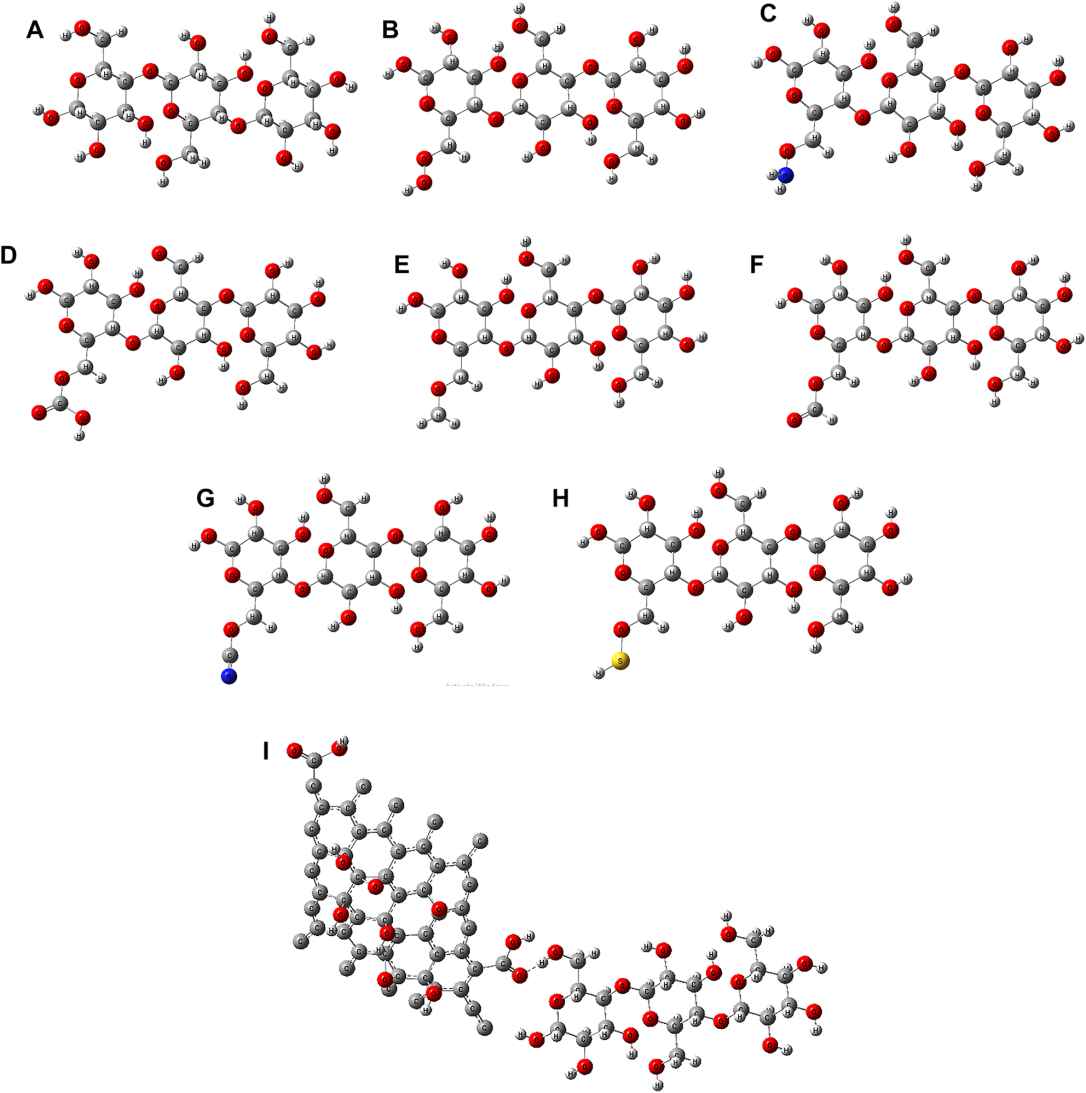
DFT: B3LYP/3–21 g** optimized structure of cellulose functionalized with various functional groups interacting through the CH 2 OH active site at the terminal position: (**a**) Cellulose, (**b**) Cellulose-OH, (**c**) Cellulose–NH 2 , (**d**) Cellulose–COOH, (**e**) Cellulose–CH 3 , (**f**) Cellulose–CHO, (**g**) Cellulose–CN, (**h**) Cellulose–SH, and (**i**) Cellulose–GO.

A model of cellulose was constructed using three cellulose units. Initially, optimization and vibrational spectra calculations were performed using the HF and DFT: B3LYP methods with various basis sets (3-21G, 6-31G, 6-311G, LANL2DZ, and LANL2MB) to compare with experimental data. The cellulose model was then considered for functionalization with the OH group in two ways: at the center and terminal units, as depicted in Fig. 1. The functionalization was envisioned to occur through the CH_2_OH group, assuming a complex interaction between the OH group and the active site of the cellulose CH_2_OH group (both the center and terminal). To determine the optimal contact site, the binding energy of these two positions was calculated. Various functional groups, including OH, NH_2_, COOH, CH_3_, CHO, CN, SH, and graphene oxide (GO) were proposed for cellulose functionalization at the optimal interaction site. Consequently, these functional groups were suggested for cellulose functionalization at the terminal position, as depicted in Fig. 2.

### Binding energy

DFT: B3LYP/3-21G** was employed to explore the optimal functionalization position along the cellulose chain (center or terminal). The binding energies for cellulose functionalized with the OH group were calculated for both the center and terminal units. Table 1presents the total energy and binding energies computed. Binding energy values are presented in a.u. and eV. Based on the current data, Cellulose-OH-Terminal has a more negative BE (-0.594 a.u. or -16.163 eV) compared to Cellulose-OH-Center (-0.564 a.u. or -15.347 eV), indicating that functionalization at the terminal unit is more energetically favorable. This trend suggests that terminal OH groups interact more strongly with cellulose, supporting greater stability. In other words, cellulose-OH-Terminal demonstrated the lowest negative binding energy, indicating it as the most favorable site for cellulose functionalization^47^.

**Table 1:** The calculated total energy (TE) and binding energy as (a.u and eV) for cellulose and functionalized cellulose with OH group through active side CH_2_OH in the two different positions center and terminal units.

Structures	TE, (a.u)	Binding Energy (a.u)	Binding Energy (eV)
OH	-75.311		
Cellulose	-1898.381		
Cellulose-OH-Center	-1973.132	-0.564	-15.347
Cellulose-OH-Terminal	-1973.103	-0.594	-16.163

### Energies and MESP of functionalized cellulose with different functional groups

To assess the impact of functionalization on cellulose activity, the Total Dipole Moment (TDM), bandgap energy, and Molecular Electrostatic Potential (MESP) were examined. Table 2 presents the calculated variations in TDM and bandgap energy (∆E) for the investigated interactions. The TDM of functionalized cellulose ranged from 4.353 Debye to 63.976 Debye for OH, NH_2_, COOH, CH_3_, CHO, CN, SH, and GO, respectively. Similarly, the bandgap energy (∆E) of functionalized cellulose ranged from 7.944 eV to 0.168 eV for OH, NH_2_, COOH, CH_3_, CHO, CN, SH, and GO, respectively. The notable reduction in ΔE for Cellulose-GO, which has the lowest bandgap energy (0.1687 eV), suggests enhanced reactivity and improved electronic conductivity. This supports previous findings by Sagadevan et al., who observed that GO-functionalized composites displayed similar improvements in conductivity and reactivity due to reduced bandgap values^48^. The concurrent increase in TDM and reduction in bandgap energy (ΔE) underscores the enhancement in both the electrical properties and structural stability of cellulose^49^. Furthermore, the substantial TDM increase in Cellulose-GO (63.975 Debye) aligns with research by Brakat et al., which demonstrated that GO functionalization significantly increased polarizability, thereby making the material more suitable for applications like flexible electronics and sensors^50^. The observed correlation between elevated TDM and lowered ΔE reflects improved electrical characteristics and stability in cellulose, in agreement with findings from other studies on functionalized polysaccharides and nanocomposites^51^. This combination of properties indicates that Cellulose-GO is highly compatible with applications requiring both high electrical responsiveness and stability, such as electronic devices and protective coatings.

**Table 2:** TDM (Debye) and HOMO-LUMO bandgap energy *ΔE* (eV) for cellulose and functionalized cellulose with different functional groups and GO interacted through the active side of cellulose CH_2_OH group in terminal position.

Structures	TDM (Debye)	∆E (eV)
Cellulose	4.353	7.944
Cellulose -OH	4.051	6.654
Cellulose -NH_2_	4.212	8.017
Cellulose -COOH	2.123	7.507
Cellulose -CH_3_	2.997	8.074
Cellulose -CHO	3.391	6.665
Cellulose -CN	6.957	6.571
Cellulose -SH	3.188	5.968
Cellulose -GO	63.975	0.168

**Figure 3:**
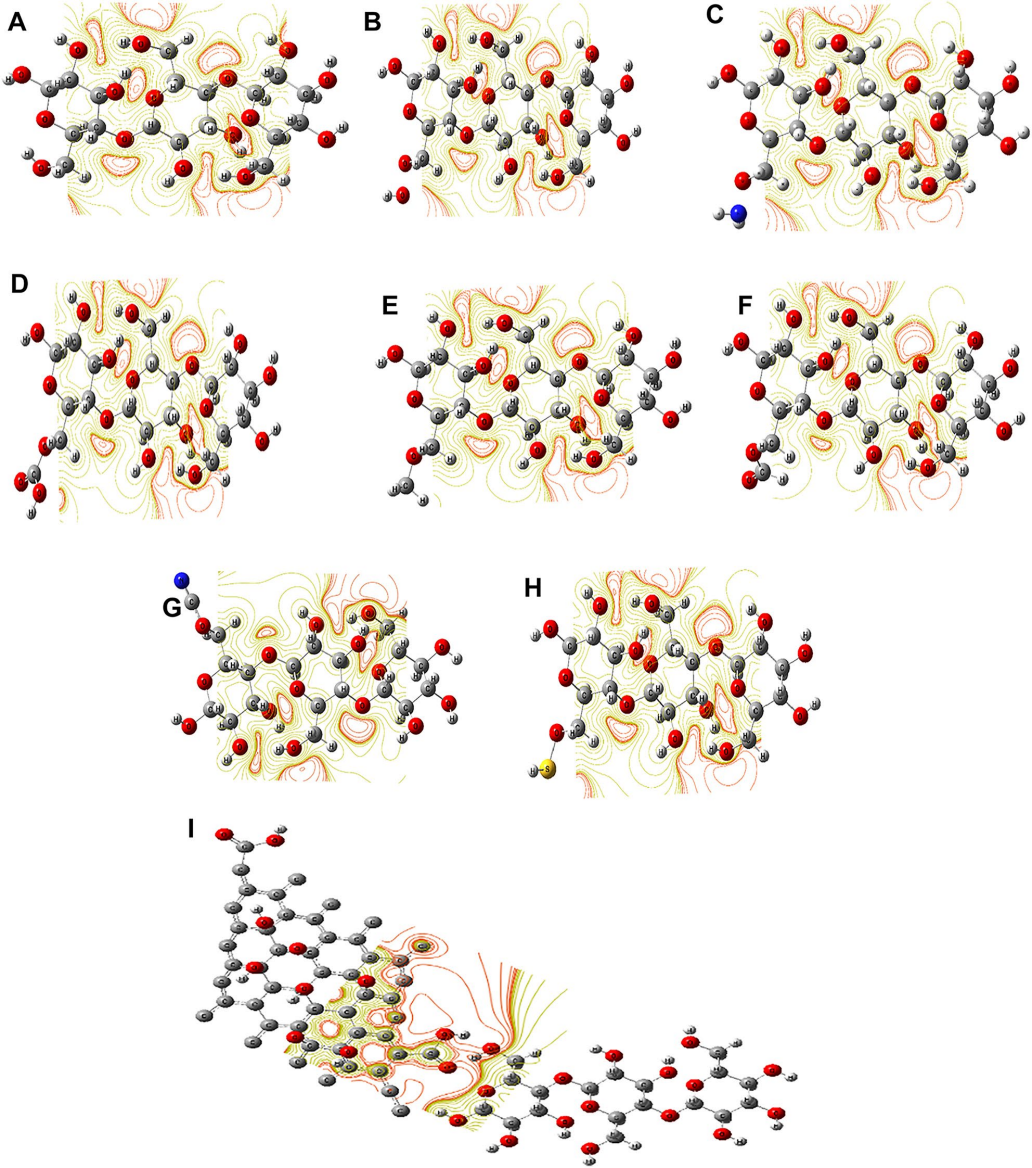
MESP of cellulose functionalized with various functional groups interacting through the terminal position using DFT: B3LYP/3–21 g**: (**a**) Cellulose, (**b**) Cellulose-OH, (**c**) Cellulose-NH 2 , (**d**) Cellulose–COOH, (**e**) Cellulose-CH 3 , (**f**) Cellulose-CHO, (**g**) Cellulose–CN, (**h**) Cellulose–SH, and (**i**) Cellulose–GO.

MESP serves as another crucial descriptor for assessing the reactivity of chemical interactions, as it delineates the impact of alterations in charge distribution on structural reactivity^52^. Figure 3 illustrates the MESP of both cellulose and functionalized cellulose with diverse functional groups and GO. Consequently, the MESP surface maps of functionalized cellulose were depicted in Fig. 3 using a spectrum of colors ranging from red (representing the highest charge area) to blue (representing the lowest charge region). The active site of cellulose was identified as the OH group of the CH_2_OH group. Upon functionalization of cellulose with various functional groups, the intensity of the red color in the regions along the polymer chain increased. The physical characteristics and reactivity of all functional groups, particularly Cellulose-GO, exhibited significant enhancement, rendering it suitable for a broad spectrum of applications. TDM, bandgap energy, and MESP were examined for cellulose models with different functional groups to scrutinize the impact of functionalization on cellulose activity.

### Inhibition parameters for cellulose-GO

As shown in Table 3, the functionalization of cellulose with graphene oxide (GO) significantly alters its electronic properties. Notably, the cellulose/GO composite exhibits a marked increase in chemical softness (σ), while its chemical hardness (η) is considerably lower than that of cellulose alone. This indicates that cellulose/GO has a greater tendency to adapt to an external electronic environment, enhancing its reactivity. Such increased softness, along with the decreased hardness, supports the composite’s ability to inhibit chemical reactions by adsorbing effectively onto metal surfaces. These properties are critical for corrosion inhibition applications, where a material’s reactivity and adsorption capacity are paramount.

**Table 3:** The global reactivity descriptors for functionalized cellulose and cellulose/GO.

Structure	LUMO	HOMO	Ionization Potential (I)	Electronic Affinity (A)	Electronic chemical potential (µ)	Chemical hardness (η)	Absolute softness (σ)	Electrophilicity index (ω)
Cellulose	1.442	-6.502	6.502	-1.442	-2.530	3.972	0.252	0.806
Cellulose -OH	0.179	-6.475	6.475	-0.179	-3.148	3.327	0.300	1.489
Cellulose -NH_2_	1.597	-6.419	6.419	-1.597	-2.410	4.008	0.249	0.725
Cellulose -COOH	0.973	-6.533	6.533	-0.973	-2.779	3.754	0.266	1.029
Cellulose -CH_3_	1.638	-6.435	6.435	-1.638	-2.398	4.037	0.248	0.712
Cellulose -CHO	0.055	-6.61	6.61	-0.055	-3.277	3.333	0.300	1.611
Cellulose -CN	0.819	-6.642	6.642	-0.819	-2.911	3.731	0.268	1.136
Cellulose -SH	-0.524	-6.492	6.492	0.524	-3.508	2.984	0.335	2.063
Cellulose -GO	-4.076	-4.244	4.244	4.076	-4.156	0.084	11.855	102.575

Furthermore, the calculated ΔN values, which represent the electron-donating ability of the inhibitors, indicate that cellulose/GO exhibits a significantly enhanced ability to donate electrons compared to pristine cellulose. This can be observed in the relative changes in the ionization potentials (I) and electronic affinities (A) across the different structures. For instance, the ionization potential of cellulose/GO is notably lower than that of cellulose, which implies a greater capacity for electron donation. In contrast, the electronic affinity remains relatively stable, suggesting that while cellulose/GO is more willing to lose electrons, it maintains its ability to stabilize additional charge. These attributes highlight the superior reactivity of cellulose/GO as an effective inhibitor, reinforcing its potential for applications requiring electron transfer and corrosion protection.

Additionally, the electrophilicity index (ω), calculated as ω = µ2/2η, confirms the unique inhibitory properties of cellulose/GO^53^. With a significantly elevated ω value, cellulose/GO demonstrates a strong ability to stabilize additional electronic charge through interactions with the metal surface. This stability, combined with its high electrophilicity, indicates that cellulose/GO not only acts as an effective inhibitor but also demonstrates enhanced stability and reactivity compared to unmodified cellulose. These attributes make cellulose/GO particularly well-suited for applications in corrosion prevention, where a balance of high softness, reactivity, and electron-donating capability is advantageous.

### Calculated DOS and PDOS

**Figure 4:**
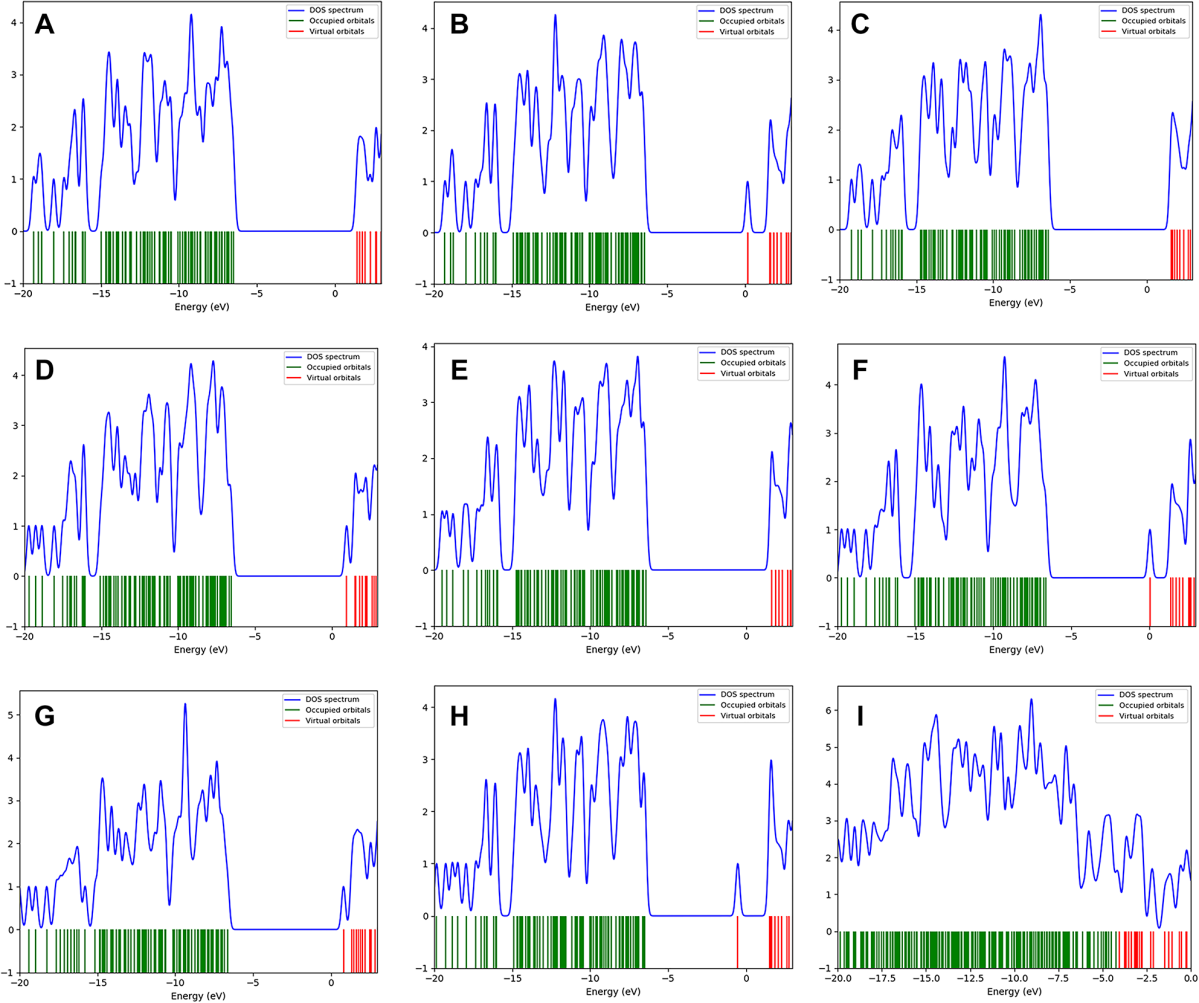
The density of states (DOS): (**a**) Cellulose, (**b**) Cellulose-OH, (**c**) Cellulose-NH 2 , (**d**) Cellulose-COOH, (**e**) Cellulose-CH 3 , (**f**) Cellulose-CHO, (**g**) Cellulose-CN, (**h**) Cellulose-SH, and (**i**) Cellulose-GO.

Figure 4 presents the Density of States (DOS) spectrum for unmodified cellulose and cellulose functionalized with various functional groups and GO. The DOS spectrum provides crucial information on the distribution of electronic states within the material, offering insights into its electronic properties. In the DOS plots, the blue line represents the density of electronic states across various energy levels. Peaks in this spectrum indicate energy levels where many electronic states are concentrated. The green lines correspond to energy levels occupied by electrons in the molecule’s ground state, which are located below the Fermi level (set to 0 eV). The red lines, on the other hand, indicate unoccupied energy levels, or virtual states, above the Fermi level. The energy region between the highest occupied molecular orbital (HOMO) and the lowest unoccupied molecular orbital (LUMO) is commonly referred to as the band gap. In the DOS spectrum, this corresponds to the energy range where no electronic states are present between the highest peak of the green lines and the lowest peak of the red lines. The size of this gap is significant for determining electrical conductivity and other electronic properties, as it defines the ease with which electrons can be excited from the HOMO to the LUMO.

Upon functionalizing cellulose with various groups and GO, the DOS spectrum reveals additional peaks, indicating new energy levels that arise from these modifications. In particular, Fig. 4h and i show states that appear close to the Fermi level. These states correspond to virtual, unoccupied states introduced by the functionalization process. Their proximity to the Fermi level suggests a reduction in the effective band gap, which can enhance the material’s electrical conductivity by allowing electrons to be more readily excited. This alteration of electronic properties due to functionalization is a key aspect for the potential use of functionalized cellulose in electronic applications. The DOS spectrum also reveals a clear band gap in most cases, indicating a semiconducting behavior. However, the additional virtual states created by the functional groups and GO suggest increased electronic interactions, which may enhance the material’s conductivity and reactivity. This information is valuable for designing cellulose-based materials with tailored electronic properties for various technological applications.

**Figure 5:**
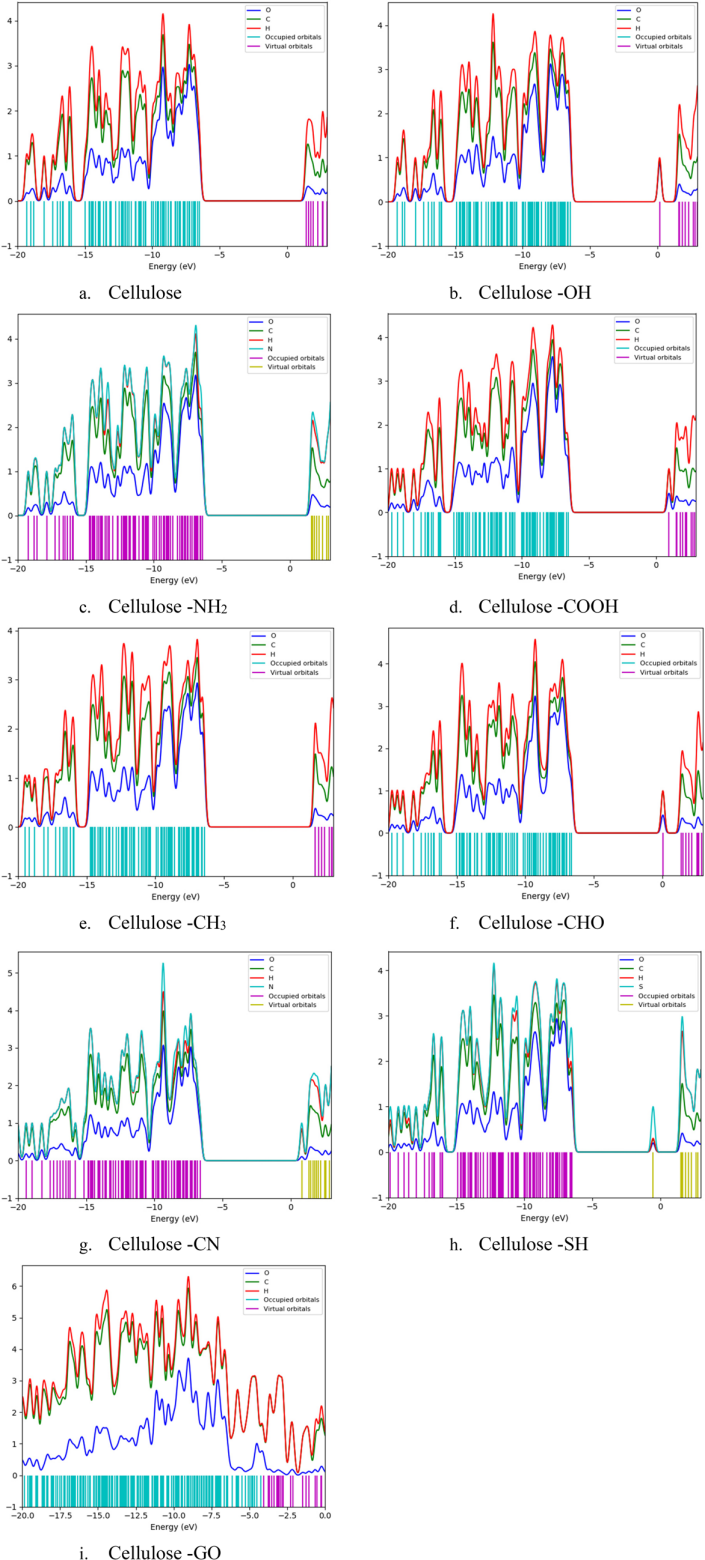
The projected density of states (PDOS): (a) Cellulose, (**b**) Cellulose-OH, (**c**) Cellulose-NH 2 , (**d**) Cellulose-COOH, (**e**) Cellulose-CH 3, (**f**) Cellulose-CHO, (**g**) Cellulose-CN, (**h**) Cellulose-SH, and (**i**) Cellulose-GO.

Figure 5 illustrates the Projected Density of States (PDOS) for cellulose and its various functionalized forms. The PDOS analysis helps in understanding the contribution of different atoms or functional groups to the electronic states of the material. Each subfigure (a to i) represents a different functionalization of cellulose, where the blue Line represents the PDOS contributed by oxygen atoms, the green line represents the PDOS contributed by carbon atoms, red line represents the PDOS contributed by hydrogen atoms, cyan sticks indicate the occupied orbitals and the magenta sticks indicate the virtual (unoccupied) orbitals. The PDOS for pure cellulose shows the distribution of electronic states contributed by its constituent atoms. Peaks in the DOS spectrum represent energy levels where a significant number of electronic states are available. The band gap can be observed as the energy range between the highest occupied and the lowest unoccupied states. Functionalization with hydroxyl groups (OH) shifts the electronic states. The presence of OH groups influences the distribution of states, particularly near the Fermi level, which might affect the band gap and reactivity. Amino groups (NH_2_) also modify the PDOS. These changes can be seen in the new peaks or shifts in existing peaks, indicating the alteration of electronic states due to NH_2_ groups. Carboxyl groups (COOH) introduce new states or modify existing ones. The PDOS indicates how COOH groups impact the overall electronic structure, potentially affecting properties like solubility and reactivity. Methyl groups (CH_3_) show their influence on the PDOS by altering the distribution of states. These changes can provide insight into how CH_3_ functionalization affects the electronic properties of cellulose. Aldehyde groups (CHO) shift the electronic states significantly. The PDOS for Cellulose-CHO can reveal how these groups impact the material’s reactivity and band gap. The influence of CN and thiol groups can also be crucial for understanding changes in electronic properties and potential applications. Graphene oxide (GO) significantly alters the PDOS of cellulose. The integration of GO introduces new states and shifts existing ones, which can drastically change the electronic properties, making Cellulose-GO composites suitable for various advanced applications.

###  IR spectrum of cellulose and its various functionalized forms

The infrared (IR) frequencies, which provide distinctive fingerprint information, have played a significant role in various chemistry fields. Consequently, the IR frequencies of cellulose were computed using HF and DFT: B3LYP methods with basis sets including 3–21 g, 6–31 g, 6–311 g, LANL2DZ, and LANL2MB, and then compared to experimental IR data. Tables 4 and 5 compare the theoretical IR frequencies of cellulose obtained from HF and DFT: B3LYP calculations with the experimental data using the mentioned basis sets. The theoretical IR data from Table 4 were utilized after each basis set was adjusted. Table 4 highlights the main characteristics of pure cellulose FTIR bands, such as the broad O-H stretching bands at 3345 cm^−1^ and C-H stretching bands around 2900 cm^−1^. Additionally, C-H and C-O vibrations were assigned to bands at 1430 and 1640 cm^−1^, respectively. The region around 1370–1340 cm^−1^ was associated with the CH_3_ umbrella mode, while the absorption bands for C-CH and C-CO of the cellulose polymer appeared at 1280 and 1320 cm^−1^. The C-O-C vibration was allocated to the band at 1160–1030 cm^−1^, and the bands for C-H and CH_2_ appeared at 895 and 615 cm^−1^, respectively^54^. A comparison of the data in Tables 4 and 5revealed that DFT: B3LYP/3–21 g yielded the closest values to the experimental data. Consequently, IR frequencies obtained using the 3–21 g** basis set were compared with those obtained using the 3–21 g basis set for improved accuracy. However, several vibrational modes obtained using DFT: B3LYP/3–21 g** were found to be in line with the experimental data, suggesting the suitability of using DFT: B3LYP/3–21 g**^55^ to examine cellulose functionalized by various functional groups and GO in close proximity to experimental results.

**Table 4:** HF calculated IR of cellulose (scaled) calculated using different basis sets including 3–21 g, 6–31 g, 6–311 g, LANL2DZ, and LANL2MB compared with cellulose IR experimental result.

Exp.	3–21 g	6–31 g	6–311 g	LANL2DZ	LANL2MB	Assignment	
615	633	633	630	631	607		**CH**_**2**_
895	872	874	877	867	931		**C-H**
1160 ~ 1030	1167- 1031	1175- 1042	1178- 1046	1168- 1035	1263- 1138		**C-O-C**
1280	1276	1279	1282	1269	1373		**C-CO**
1320	1324	1328	1334	1318	1438		**C-CH**
1370 ~ 1340	1377- 1345	1381- 1346	1383- 1350	1373- 1335	1494- 1456		**Split CH**_**3**_ **umbrella mode**
1430	1430	1436	1432	1428	1586		**C-H**
1640	-	-	-	-	-		**C-O**
2900	2990	2941	2945	2960	3166		**CH Sym. Str.**
3345	3342	3512	3563	3537	3800		**O-H Stretching**

**Table 5:** DFT: B3LYP calculated IR of cellulose (scaled) calculated using different basis sets including 3–21 g, 6–31 g, 6–311 g, LANL2DZ, LANL2MB, and 3–21 g** compared with cellulose IR experimental result.

Exp.	3–21 g	6–31 g	6–311 g	LANL2DZ	LANL2MB	3–21 g**	Assignments
615	637	629	629	611	611	635	**CH**_**2**_
895	890	716	723	850	851	849	**C-H**
1160 ~ 1030	1122- 1029	1132- 1029	1131- 1028	1167- 1092	1167- 1092	1117- 1022	**C-O-C**
1280	1278	1273	1274	1311	1311	1281	**C-CO**
1320	1324	1308	1312	1375	1375	1320	**C-CH**
1370 ~ 1340	1374- 1343	1370- 1339	1371- 1339	1422- 1396	1422- 1396	1371- 1339	**Split CH**_**3**_ **umbrella mode**
1430	1418	1417	1393	1562	1595	1417	**C-H**
1640	-	-	-	-	-	-	**C-O**
2900	2943	2956	2934	3105	3105	3021	**CH Sym-stretching**
3345	3388	3503	3551	3537	3537	3619	**O-H Stretch**

**Figure 6:**
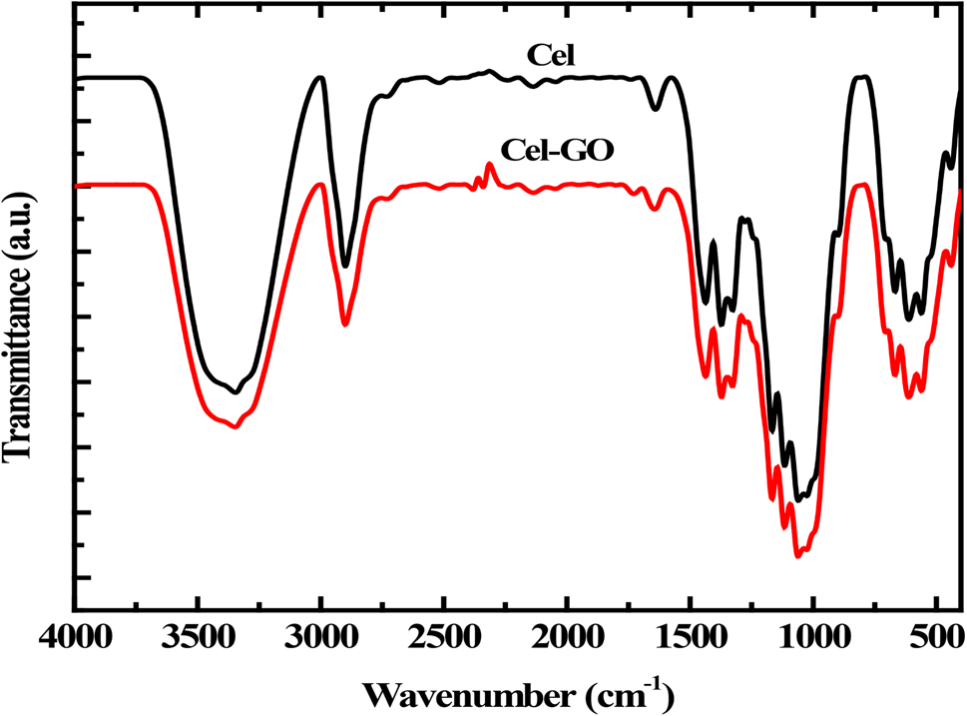
FTIR Transmittance spectra of cellulose and cellulose-GO.

Experimental FTIR spectroscopy of cellulose-GO film is depicted in Fig. 6 and outlined in Table 6. The characteristic bands of cellulose encompass a broadband predicted at 3325 cm^−1^, attributed to the OH groups of both cellulose and GO. Upon the synthesis of cellulose/GO composite via the OH group of CH_2_OH, a new band representing the GO (COOH) group emerged at 1710 cm cm^−1^. The interaction between cellulose and GO through the CH_2_OH group caused a shift of the entire cellulose band to a lower wavenumber. It has been previously established that IR absorption occurs due to the interaction between the IR electric field vector and the molecular dipole transition moments, which are associated with molecular vibrations. Absorption is maximized when the electric field vector and the dipole transition moment are parallel^56^. Applying this principle to the experimental FTIR spectra, variations in the IR-absorbance intensity, particularly in C-O at 1620 cm^−1^as Bronsted acid as previously discussed, are evident^57^. These variations are expected to be more pronounced in cellulose without graphene, in correlation with the dipole moment values obtained from modeling, as shown in Table 2.

**Table 6:** Band assignment of FTIR result of cellulose and Cellulose-GO.

Cellulose-GO	Assignments
605	**CH**_**2**_
875	**C-H**
1140 ~ 1010	**C-O-C**
1280	**C-CO**
1306	**C-CH**
1350 ~ 1320	**Split CH**_**3**_ **umbrella mode**
1410	**C-H**
1620	**C-O**
1710	**COOH of GO**
2880	**CH Sym. Str.**
3325	**O-H Stretching**

## Conclusions

In conclusion, this study examined the functionalization of cellulose with various functional groups and graphene oxide (GO) to enhance its properties for diverse applications. Through computational modeling and experimental analyses, we investigated the vibrational spectra, electronic properties, and reactivity of cellulose and its functionalized derivatives. Our findings indicate that DFT: B3LYP/3–21 g** emerged as the optimal method for predicting the vibrational frequencies of cellulose, while functionalization with GO at the terminal position exhibited the most favorable binding energy and stability. The DOS and PDOS analysis provides detailed insights into how different functional groups affect the electronic structure of cellulose. By comparing the DOS and PDOS of pure and functionalized cellulose, one can understand the influence of each functional group on the material’s electronic properties. Furthermore, the study examined the interaction between cellulose and GO via the CH_2_OH group, revealing a notable shift in the cellulose band to a lower wavenumber. This observation aligns with theoretical principles regarding IR absorption and molecular dipole moments, as elucidated in our discussion. The experimental FTIR spectra further highlighted variations in IR-absorbance intensity, particularly in the C-O region at 1620 cm^[-1^, confirming the impact of functionalization on cellulose’s structural characteristics. In fact, this study provides a comprehensive investigation into the electronic properties and stability of cellulose and its functionalized derivatives, particularly in the context of their applications in advanced materials and nanocomposites. Understanding the electronic characteristics, such as binding energy, chemical reactivity, and density of states, is crucial for the development of cellulose-based materials in fields like flexible electronics, sensors, and bioengineering. By elucidating these properties, our work aims to contribute valuable insights into the design and optimization of cellulose derivatives for enhanced performance in various technological applications.

## Data Availability

The data that support the findings of this study are available from the corresponding author upon reasonable request.
